# Vulnerable Narcissism Modulates Early Neural Processing of Verbal Violence in Women: An ERP Study

**DOI:** 10.3390/bs16020270

**Published:** 2026-02-12

**Authors:** Qianglong Wang, Ping Song, Yongxiang Hu, Rongbao Li

**Affiliations:** 1 College of Foreign Languages, Fujian Normal University, Fuzhou 350007, China; qlwangpsy@xjnu.edu.cn; 2 School of Psychology, Xinjiang Normal University, Urumqi 830017, China; 3 Xinjiang Key Laboratory of Mental Development and Learning Science, Urumqi 830017, China; 4 Institute of Investigation and Counter-Terrorism, Criminal Investigation Police University of China, Shenyang 110854, China; songping@cipuc.edu.cn; 5 College of Foreign Languages, Fujian Polytechnic Normal University, Fuzhou 350300, China; huyx@fpnu.edu.cn

**Keywords:** narcissism, women, verbal violence, ERP

## Abstract

This study examined how narcissistic traits influence women’s cognitive processing of verbal violence. Using a lexical decision task, an emotional Stroop task, and event-related potentials, we analyzed neural responses to violent versus neutral words in 70 women. Behaviorally, while narcissism showed no significant impact on performance in the Lexical Decision Task, a specific interference effect emerged in the emotional Stroop task, where higher narcissistic vulnerability predicted reduced accuracy for violent words relative to neutral ones. Notably, ERP results revealed a consistent pattern across both tasks: higher PNI total scores significantly predicted reduced amplitudes of early components, specifically the N170 and P2. Furthermore, in the emotional Stroop task, the vulnerability dimension emerged as a significant predictor of reduced EPN and P2 amplitudes. These findings suggest that when exposed to verbal violence, narcissistic women exhibit attenuated early evaluation and attentional allocation. This reflects a preemptive cognitive avoidance strategy used to protect the self-concept, driven primarily by a general narcissistic defensive pattern that manifests most acutely in vulnerable traits under high-interference conditions.

## 1. Introduction

Verbal violence is a form of psychological harm characterized by the use of insulting, obscene, and vulgar language to attack an individual’s dignity and worth (Aytac et al., 2011). While societal status has improved, women continue to face structural vulnerabilities—especially on digital platforms—making them prone to verbal violence targeting their appearance and abilities. Notably, the psychological impact of such aggression may be modulated by how individuals interpret social threats within their specific socio-cultural contexts.

Consistent with this socio-cultural vulnerability, extensive research suggests that women, on average, tend to exhibit heightened emotional reactivity and a greater propensity for internalizing negative affect when exposed to interpersonal affect (Nolen-Hoeksema, 2012; Stevens & Hamann, 2012). This may increase their susceptibility to emotional distress when encountering verbal violence. However, the degree of this vulnerability and the subsequent defensive response are critically shaped by an individual’s underlying personality architecture. Among these, narcissism—a trait characterized by an inflated yet fragile self-concept—has become increasingly prevalent in modern social contexts (Twenge & Campbell, 2009). Therefore, how do women exhibiting narcissistic traits—individuals characterized by both heightened sensitivity to self-worth evaluations and sensitivity to threatening language—cope with verbal violence? Answering this requires an understanding that narcissism manifests distinctly across genders. It is a complex personality trait characterized by egocentrism, a sense of entitlement, and feelings of superiority, typically manifesting in a combination of grandiose and vulnerable features to varying degrees (Krizan & Herlache, 2017). Grandiose features commonly include immodesty, aggressiveness, and arrogance; vulnerable features are often marked by defensiveness, low self-esteem, and hypersensitivity to criticism. While individuals with narcissistic traits typically exhibit a blend of both, research indicates significant gender differences in their relative prominence (Green et al., 2021). Specifically, male narcissists are predominantly characterized by grandiose features, whereas female narcissists display more prominent vulnerable features (Green et al., 2019, 2020). However, a significant limitation of prior research is its reliance on tools (e.g., the NPI) that primarily assess grandiose traits (Blinkhorn et al., 2019). This methodological bias has potentially led to findings that overestimate narcissism in males while overlooking the distinct, vulnerable profile more commonly observed in females.

Thus, shifting the focus toward female narcissism is essential to uncovering the distinct cognitive-affective processing styles associated with the vulnerable dimension. Unlike the grandiose narcissist, who often employs “narcissistic reactance” or overt aggression to deflect threats, the vulnerable narcissist—rooted in a ‘brittle sense of self’ (Grijalva et al., 2015)—is characterized by hypersensitivity to social evaluation and deep-seated feelings of inadequacy (Pincus & Lukowitsky, 2010). This profile suggests that their defense mechanisms are more internalized and cognitive-heavy, likely characterized by a “vigilance–avoidance” pattern rather than externalized hostility. By focusing on this specific demographic, the current study moves beyond the well-documented “aggressive outburst” model of narcissism to uncover latent defensive processing—such as cognitive devaluation or attentional bias—that occurs during the early stages of information encoding. Without this targeted focus, the field’s understanding remains biased toward male-typical grandiose manifestations, overlooking the nuanced self-protection strategies employed by individuals with a more vulnerable profile (Pincus & Lukowitsky, 2010).

Narcissistic traits render individuals highly sensitive to external evaluations, triggering defensive coping strategies—such as denial or devaluation—to protect a fragile self-image (Atlas & Them, 2008; Hepper et al., 2022). However, as overt manifestations often mask concurrent internal psychological processes (Z. Yang et al., 2018), externally observed responses may fail to capture the underlying defensive mechanisms. Examining the cognitive processing of verbal violence offers a novel perspective to reveal whether these defenses emerge as early as the information-encoding stage.

This cognitive lens is particularly critical because verbal violence differs qualitatively from the task-oriented negative feedback studied previously (Barry et al., 2006). Unlike performance-based critiques, verbal violence carries higher aggression potential and constitutes a direct assault on identity and human dignity (X. Zhang et al., 2024). For narcissistic women, such aggression does not merely signal a failure to meet standards; it inflicts a “narcissistic injury” that threatens the very core of their unstable self-concept (Pincus & Lukowitsky, 2010).

The theoretical foundation for this study is further strengthened by evidence of domain-specific attentional biases. Research using the dot-probe task has demonstrated that narcissists exhibit significant vigilance toward failure-related words, yet surprisingly, they do not show the same bias toward general interpersonal rejection words (Gu et al., 2013). This suggests that their cognitive system is highly “tuned” to specific ego-threats rather than generic negative feedback. Parallel to these passive processing biases, the relationship between narcissism and language extends beyond reception to active, defensive linguistic styles. Grandiose narcissism is consistently associated with an increased use of swear words and a strategic avoidance of words expressing anxiety or fear (Holtzman & Mehl, 2011). This “linguistic masking”—where aggression is used to suppress underlying vulnerability—is a hallmark of narcissistic self-regulation that remains stable even into late life (S. Zhang et al., 2023).

Despite these significant insights, existing linguistic research remains heavily skewed toward the grandiose dimension and its use of “best words” for self-promotion (Underberg et al., 2020). There remains a critical empirical gap regarding the vulnerable dimension, which is more prominent in women and centers on an internalized “brittle self.” To address this, the current study investigates how these individuals cognitively encode and regulate responses to verbal violence.

To accurately capture these nuances, the Pathological Narcissism Inventory (PNI) will be used to assess pathological narcissism in female participants (Pincus, 2013). Unlike the previously prevalent Narcissistic Personality Inventory (NPI), which primarily captures grandiose features (e.g., leadership, authority), the PNI provides a more comprehensive assessment by incorporating dimensions of narcissistic vulnerability, such as contingent self-esteem and devaluing, which are central to the “brittle self-concept” in women (Grijalva et al., 2015; Pincus & Lukowitsky, 2010).

To examine the cognitive processing of verbal violence, this study employs two complementary experimental paradigms: a lexical decision task (LDT) and an emotional Stroop task. The choice of the LDT is grounded in its ability to measure the automaticity of semantic activation. Research has demonstrated that narcissistic traits are associated with specific attentional biases and hypersensitivity to ego-threatening linguistic stimuli (Gu et al., 2013). By requiring rapid identification of word versus non-word strings, the LDT allows us to capture the immediate neural response as narcissistic individuals encode verbal violence, reflecting the degree to which these aggressive stimuli penetrate their cognitive defense system at an early stage (Z. Yang et al., 2018).

Complementing the LDT’s focus on initial encoding, the emotional Stroop task is utilized to assess the subsequent inhibitory control and emotional interference caused by verbal violence. According to the self-regulatory model of narcissism (Pincus & Lukowitsky, 2010), narcissistic individuals often experience significant affective dysregulation when encountering social threats. The emotional Stroop task—where participants must name the ink color while ignoring the word’s offensive meaning—measures the extent to which verbal violence captures attentional resources and impairs cognitive performance (H. Zhang et al., 2018). While the LDT focuses on initial semantic access, the emotional Stroop task probes the persistent interference effect and the cognitive effort required to suppress the emotional impact of the threat. Together, these tasks provide a multi-dimensional view of how narcissistic women cognitively navigate and defend against identity-based aggression.

However, while these behavioral paradigms reveal the outcomes of cognitive interference, they cannot fully disentangle the instantaneous neural stages where these defenses are deployed. While research on verbal violence has predominantly relied on questionnaires and self-reports (Guay et al., 2014; Rosenthal et al., 2018), these methods often fail to capture the real-time cognitive dynamics of aggression processing. Event-related potentials (ERPs) provide the millisecond-level resolution necessary to disentangle these processes, yet their application in this field remains scarce (Wang et al., 2025). By utilizing ERPs, the current study aims to determine the temporal sequence of brain activity during the processing of verbal violence in women with narcissistic traits. Specifically, we seek to clarify whether their defensive response occurs at the early stage of initial sensory perception or during the later stage of semantic integration. This approach offers a level of analysis that may reveal mechanisms distinct from the task-oriented feedback responses documented in the previous literature.

During the experiment, violent words will be presented visually, and the temporal dynamics of their processing will be captured using four established ERP components that serve as neural indices of distinct cognitive stages.

First, the N170 (140–200 ms) reflects early structural analysis of orthographic features (Simon et al., 2007). In emotional research, N170 amplitudes are significantly enhanced by emotional stimuli compared to neutral ones, indicating early sensitivity to affective salience (D. Zhang et al., 2014). Second, the P2 (150–250 ms) is associated with rapid visual feature extraction and initial stimulus screening (Coch & Meade, 2016). Notably, negative words typically produce larger P2 amplitudes, reflecting a rapid detection of potential threats (Ding et al., 2016; González-Villar et al., 2014).

Following these early sensory phases, the EPN (200–300 ms) indexes automatic emotional attention and the transition into early semantic extraction (Kissler et al., 2009). Finally, the N400 (300–500 ms) serves as a marker for deeper semantic integration and the resolution of cognitive conflict; reduced N400 amplitudes indicate facilitated access to a word’s meaning within an existing mental schema (Kutas & Federmeier, 2011).

Critically, these components are no longer limited to linguistic research; they have been increasingly utilized in personality psychology to capture individual differences in social-cognitive processing (Krasowski et al., 2021; Ku et al., 2020; Mück et al., 2020). By monitoring this sequence of ERPs, we can pinpoint whether the defensive mechanisms of narcissistic women alter the processing of verbal violence at the level of initial perception (N170/P2), automatic attention (EPN), or intentional semantic evaluation (N400) Building on the Dynamic Self-Regulatory Processing Model (Morf & Rhodewalt, 2001), we propose that narcissistic individuals employ “defensive filtering” to mitigate the impact of ego-threats. While behavioral evidence suggests that narcissists visually avoid negative information (Horvath & Morf, 2009), it remains unclear when and how this defense operates during the actual processing of verbal aggression.

To enhance the transparency of our investigation, the present study addresses the following Research Question: Does the defensive mechanism of narcissistic women against verbal violence occur at the early stage of initial perception or during the later stage of conscious interpretation? Based on the theory of defensive avoidance (Pincus & Lukowitsky, 2010), we test the following Hypotheses:

**H1.**  *We hypothesize that women with higher narcissistic traits will show smaller N170 and P2 amplitudes in response to violent words compared to neutral words. This would indicate that their defensive “filter” is activated at the very beginning of stimulus perception, leading to reduced sensitivity to aggressive cues*.

**H2.**  *We predict that narcissistic traits will be associated with reduced EPN amplitudes for violent words. This reflects a diminished automatic capture of emotional information, suggesting that these individuals cognitively “bypass” the emotional salience of verbal violence*.

**H3.**  *We hypothesize that N400 amplitudes will be significantly lower during the processing of verbal violence in narcissistic women. This would suggest a defensive attenuation of the word’s meaning during the final stage of semantic integration*.

**H4.**  *Pathological narcissism scores (specifically the vulnerability dimension) will be negatively correlated with the amplitudes of all four ERP components (N170, P2, EPN, and N400) elicited by violent words. This demonstrates that as narcissistic traits increase, the brain’s neural responsiveness to verbal aggression systematically decreases*.

## 2. Materials and Methods

### 2.1. Participants

Sample size estimation was performed using G*Power 3.1.9.4. Based on the within-subjects design and planned repeated-measures ANOVA with four measurement conditions, a medium effect size (f = 0.25), α = 0.05, and power = 0.95, the analysis indicated a minimum requirement of 36 participants.

Seventy-four female university students (M age = 22.7 years, SD = 1.96; range: 18–30 years) were recruited. All participants met the following criteria: (a) normal/corrected-to-normal vision, (b) right-handedness, (c) no history of neurological disorders or psychiatric conditions. Prior to experiment, participants provided written informed consent and received monetary compensation after the study. Four participants were excluded due to excessive EEG artifacts (>50% trials rejected), yielding a final sample of N = 70. The study was approved by the Academic Committee of the Department of Applied Linguistics at Fujian Normal University (NO. 2025012).

An a priori sensitivity power analysis was conducted using G*Power 3.1.9.4. With a final sample of N = 74, an alpha level of 0.05, and a standard power of 0.80, the study was sufficiently powered to detect a minimum effect size of f2 = 0.108 for the interaction between the continuous predictor (PNI scores) and the categorical factor (Word Type).

### 2.2. Materials

#### 2.2.1. Pathological Narcissism Inventory

The Pathological Narcissism Inventory (PNI) is a 52-item instrument assessing two core dimensions of pathological narcissism: grandiosity and vulnerability (Pincus, 2013). It comprises seven subscales: Contingent Self-Esteem, Exploitativeness, Self-Sacrificing Self-Enhancement, Grandiose Fantasy, Self-Devaluation, Entitlement Rage, and Hiding the Self. Responses are recorded on a 6-point Likert scale (0 = Not at all like me to 5 = Very much like me). Consistent with the theoretical model, the grandiosity dimension comprises Entitlement Rage, Exploitativeness, Grandiose Fantasy, and Self-Sacrificing Self-Enhancement, whereas vulnerability includes Contingent Self-Esteem, Hiding the Self, and Self-Devaluation. In this sample, the Cronbach’s α coefficient for the PNI was 0.94.

#### 2.2.2. Violent Words

Sixty violent words and sixty neutral words were selected from the Chinese Lexical Database (Sun et al., 2018). Violent words consisted primarily of nouns and adjectives denigrating appearance, ability, intelligence, or personality. All violent words were two-character Chinese words, controlling for stroke count. Thirty independent raters (19 female, 11 male; aged 18–32 years) evaluated word aggression using a 9-point Likert scale (1 = Extremely aggressive, 9 = Extremely non-aggressive). Results indicated significantly lower valence ratings for violent words (M = 3.42, SD = 0.22) relative to neutral words (M = 5.74, SD = 0.06), t = 86.39, *p* < 0.001, and Cohen’s d = 9.66. Violent words (M = 4.53, SD = 0.30) also had higher arousal ratings than neutral words (M = 2.30, SD = 4.45), t = 3.13, *p* = 0.002, and Cohen’s d = 0.70.

### 2.3. Procedure

The experiment was conducted in a sound-attenuated, electromagnetically shielded chamber to minimize interference with ERP data collection. Participants sat 60 cm from the screen. After being seated, they studied written instructions for both tasks to ensure full understanding of the requirements and procedures. Prior to the formal experiment, they completed 5 practice trials per task to familiarize themselves with the operations. The two tasks were programmed using E-Prime 2.0 and presented on a 27-inch LCD monitor (1920 × 1080 resolution, 60 Hz refresh rate).

**Figure 1:**
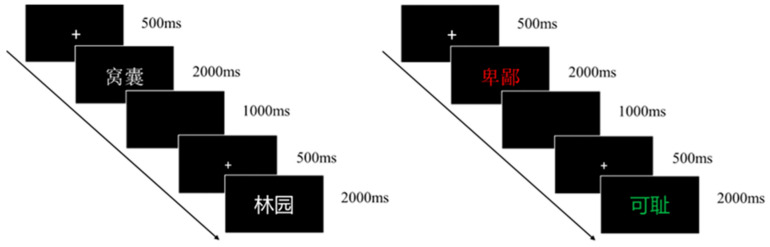
Flowcharts of the lexical decision task (left) and the emotional Stroop task (right). Note. English glosses for Chinese violent words: Spineless (窝囊); Despicable (卑鄙); Shameful (可耻ǐ); Garden (林园).

Lexical Decision Task (Figure 1): at the start of each trial, a fixation cross (“+”) was presented at the center of the screen for 500 ms, followed by the random presentation of a word that remained on the screen for 2000 ms. After an interval of 1000 ms, the next trial began. Participants were required to quickly judge the type of the word during its presentation and respond by pressing the corresponding key (“F” for verbal violence words, “J” for neutral words). The task was divided into 2 blocks, comprising a total of 120 trials.

Emotional Stroop Task (Figure 1): at the start of each trial, a fixation cross (“+”) was presented at the center of the screen for 500 ms, followed by a colored word that remained visible for 2000 ms. After an interval of 1000 ms, the next trial began. Participants received instructions to ignore the word’s meaning and rapidly judge its font color during its presentation, responding by pressing the key corresponding to that color (“Z” for red, “V” for blue, “M” for green). The task was divided into 2 blocks with a 2 min break between blocks, totaling 120 trials. The distribution of the three colors was equal across both violent and neutral words.

Half of the participants completed the lexical decision task first, followed by the emotional Stroop task; the other half completed the tasks in the reverse order. Both tasks were divided into 2 blocks each, with a 2-min break between blocks.

### 2.4. EEG Recording and Analysis

EEG signals were recorded using a 128-channel HydroCel Geodesic Sensor Net (Electrical Geodesics Inc., Eugene, OR, USA) with a sampling rate of 250 Hz, referenced to the vertex electrode (Cz). Electrode positions corresponded to the international 10–10 system layout, with impedances maintained below 5 kΩ. A detailed layout of the electrode positions is shown in Supplementary Figure S1.

Offline analysis was performed using EEGLAB (2024.1) and ERPLAB (12.01) toolboxes (Delorme & Makeig, 2004; Lopez-Calderon & Luck, 2014) in MATLAB R2020b. The continuous EEG data were filtered with a 0.1–30 Hz zero-phase bandpass filter and re-referenced to the average of all electrodes. ICA was then applied to the continuous data to correct eye-blink artifacts. EEG epochs were segmented from −200 ms to 1000 ms relative to stimulus onset. Epochs with amplitudes exceeding ±100 µV were excluded, and baseline correction was applied using the −200 ms to 0 ms time window. For the lexical decision and emotional Stroop tasks, 96.5% and 95% of trials were retained, respectively.

Based on previous literature (Kanske & Kotz, 2007; Kissler et al., 2009; Simon et al., 2007) and visual inspection of waveforms, the following electrode clusters and time windows were selected for analysis:

Lexical Decision Task: N170 (150–200 ms): Electrodes [64, 65, 68, 69, 89, 90, 94, 95]; EPN (216–268 ms): Electrodes [64, 65, 68, 69, 89, 90, 94, 95]; P2 (170–260 ms): Electrodes [129, 6, 7, 13, 31, 80, 106, 112]; N400 (280–420 ms): Electrodes [7, 30, 31, 55, 80, 105, 106].

Emotional Stroop Task: N170 (150–250 ms): Electrodes [64, 65, 69, 89, 90, 95]; EPN (220–300 ms): Electrodes [57, 58, 64, 65, 95, 96, 99, 100]; P2 (150–250 ms): Electrodes [5, 6, 7, 12, 13, 106, 112]; N400 (300–450 ms): Electrodes [9, 10, 11, 15, 16, 18, 22].

Both tasks employed a within-subjects design with narcissistic traits scores (continuous variable) as a core predictor. PNI scores were retained to maximize data information and statistical power (Altman & Royston, 2006). Lexical Decision Task: Incorporated a 2-factor design: PNI scores × Word Type (violent/neutral). Emotional Stroop Task: Utilized a 3-factor design: PNI scores × Word Type (violent/neutral) × Color (red/green/blue). Dependent variables comprised behavioral measures (accuracy, reaction time) and ERP mean amplitudes (N170, EPN, P2, N400).

Data were analyzed using linear mixed-effects models (LMM) in JASP (Version 0.95.3). For both behavioral performance (accuracy and reaction times) and ERP components (N170, EPN, P2, and N400), PNI scores (a continuous variable) and Word Type (categorical: violent vs. neutral) were entered as fixed effects, along with their interaction term. To account for the hierarchical structure of the data and individual differences, participants were included as a random intercept.

Following standard practices for modeling interactions with continuous predictors, PNI score was mean-centered within the software to enhance the interpretability of the results and reduce multicollinearity. The Satterthwaite approximation was employed to estimate degrees of freedom and *p*-values. For any significant interactions involving PNI score, simple slope tests were conducted to further examine the predictive effect of PNI at each level of Word Type.

## 3. Results

### 3.1. PNI Score

The PNI total scores in our sample ranged from 101 to 247. Descriptive statistics for the total score and each subscale are summarized in Table 1. Shapiro–Wilk normality tests indicated that, with the exception of the Hiding the Self and Narcissistic Grandiosity dimensions, the total score and all other dimensions followed a normal distribution (*p*s > 0.05).

### 3.2. Behavioral Results

#### 3.2.1. Lexical Decision Task

Accuracy: The main effect of PNI score was not significant, F (1, 136) = 2.53, *p* = 0.11, and the main effect of word type was non-significant, F (1, 136) = 1.85, *p* = 0.18. Their interaction was also non-significant, F (1, 68) = 3.51, *p* = 0.06.

Reaction Time: Results showed no significant main effect of PNI score, F (1, 68) = 0.003, *p* = 0.96, or word type, F (1, 68) = 2.26, *p* = 0.14. The interaction between PNI score and word type did not reach significance, F (1, 68) = 3.46, *p* = 0.07.

#### 3.2.2. Emotional Stroop Task

Accuracy: Linear Mixed Models revealed that the main effect of PNI score was not significant, F (1, 68) = 0.09, *p* = 0.76, nor was the main effect of word type, F (1, 68) = 3.33, *p* = 0.07. A significant interaction between PNI and word type was found, F (1, 68) = 4.40, *p* = 0.04. However, simple slope tests demonstrated that the predictive effect of PNI score was not significant for either violent words or neutral words (all *p*s > 0.05). To further clarify this interaction, we calculated the accuracy difference scores (violent minus neutral) and conducted correlation analyses. Results showed that the accuracy difference score was significantly negatively correlated with the PNI total score (r = −0.25, *p* = 0.04) and the narcissistic vulnerability dimension (r = −0.25, *p* = 0.034), while the correlation with grandiosity did not reach significance (r = −0.20, *p* = 0.096).

Reaction Time: The main effect of PNI score was not significant, F (1, 68) = 0.16, *p* = 0.69, and the main effect of word type was also non-significant, F (1, 68) = 0.36, *p* = 0.55. The interaction between the two was not significant, F (1, 68) = 0.39, *p* = 0.54.

### 3.3. ERP Results

#### 3.3.1. Lexical Decision Task

Linear mixed models were used to examine the effects of PNI score, word type (violent/neutral), and their interaction on four ERP components (Table 2).

For the N170 component, a significant main effect of PNI score was observed. The main effect of word type and the interaction were not significant.

For the EPN component, neither the main effects of PNI score and word type, nor their interaction, reached statistical significance.

For the P2 component, significant main effects were found for both PNI scores and word type. Higher PNI scores predicted a significant decrease in P2 amplitude. Furthermore, a significant PNI scores by word type interaction was found, To decompose the significant interaction on the P2 component, a simple slopes analysis was performed. The effect of PNI scores was significant only for violent words, demonstrating a significant negative slope (Slope = −0.014, SE = 0.005, z = −2.90, *p* = 0.004, 95% CI [−0.023, −0.004]). In contrast, the slope for neutral words was non-significant (Slope = −0.006, SE = 0.005, z = −1.16, *p* = 0.246, 95% CI [−0.015, 0.004]).

For the N400 component, a significant main effect of word type was found, but the main effect of PNI scores and the interaction were not significant.

#### 3.3.2. Emotional Stroop Task

Linear mixed models were used to examine the effects of PNI scores, word type, and their interaction on ERP components within the Emotional Stroop task (Table 3).

For the N170 component, no significant main effects or interaction were observed.

For the EPN component, a significant main effect of PNI scores was found. The main effect of word type and the interaction were not significant.

For the P2 component, a significant main effect of PNI scores was also identified, showing that higher PNI scores predicted a decrease in P2 amplitude. Neither the main effect of word type nor the interaction reached significance. Finally, for the N400 component, no significant effects were found.

### 3.4. Regression Analysis for ERP Results

#### 3.4.1. Correlation Results

**Figure 2:**
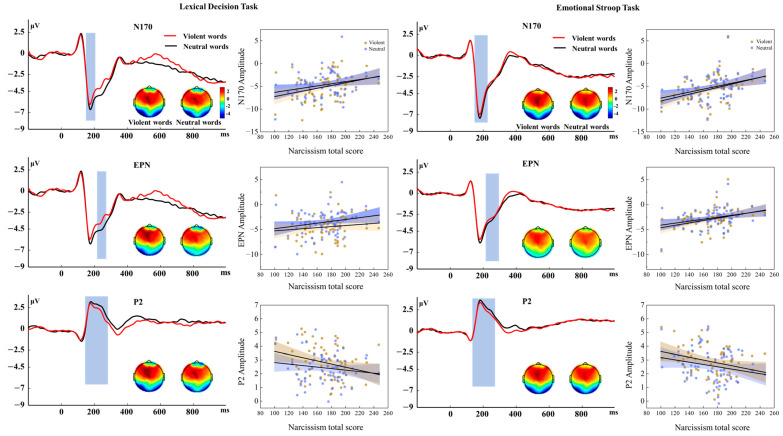
Grand–average waveforms and topographic maps for N170, EPN, and P2 components elicited by violent and neutral words, with scatterplots showing correlations between component amplitudes and PNI scores.

To assess how narcissistic traits predict ERP components during verbal violence processing, Pearson correlation analyses were conducted (Figure 2). Narcissism total scores and the vulnerability dimension showed significant negative correlations with early components (N170, EPN, P2) for both violent and neutral words (rs = 0.32 to −0.33, *p*s < 0.05; Table 4). In contrast, the grandiosity dimension correlated significantly with only N170 and P2 (violent words), while no significant associations emerged for EPN and N400 (Table 4).

Furthermore, to isolate the neural responses specifically elicited by verbal violence, we analyzed the correlations between PNI scores and the ERP difference waves (violent minus neutral). Notably, in the LDT, the P2 difference wave showed significant negative correlations with both PNI total scores (r = −0.24, *p* < 0.05) and the grandiosity dimension (r = −0.29, *p* < 0.05). This indicates that higher levels of narcissism specifically predict a greater reduction in early neural orientation toward violent words relative to the neutral words. No other significant correlations emerged for difference waves in other components or tasks.

#### 3.4.2. Regression Results

To further clarify the impact of narcissism, regression analyses examined the predictability of the dual-dimension narcissism model (grandiosity and vulnerability) and the narcissism total score model on N170, EPN, and P2 amplitudes. The results showed that:

1. The narcissism total score model

Violent words: PNI scores significantly negatively predicted amplitude reductions in N170 and P2 components in both tasks; significant prediction of EPN occurred only in the emotional Stroop task (Table 5).

PNI scores negatively predicted EPN amplitudes of neutral words in emotional Stroop task: b(SE) = 0.020(0.007), 95% CI [0.005, 0.034], t = 3.69, *p* = 0.009, β = 0.310.

2. The dual-dimension narcissism model

The vulnerability dimension negatively predicted EPN and P2 amplitudes for violent words in the emotional Stroop task (Table 5).

It also negatively predicted EPN amplitudes for neutral words in the emotional Stroop task: b (SE) = 0.040(0.017), 95% CI [0.006, 0.075], t = 2.32, *p* = 0.02, β = 0.425.

## 4. Discussion

This study utilized ERPs to investigate the temporal dynamics of how women with narcissistic traits process verbal violence. Behaviorally, narcissism showed no consistent significant impact on reaction time or accuracy across the lexical decision and emotional Stroop tasks. This suggests that the impact of verbal violence does not manifest in overt performance, potentially because the cognitive response occurs before the stage of behavioral output. However, the observed dissociation between early neural attenuation and stable reaction times warrants further consideration. This lack of behavioral slowing suggests that the “neural defense” operates as an efficient gating mechanism. By reducing neural engagement with threatening stimuli at an early stage, narcissistic individuals may effectively “mute” the emotional impact of verbal violence, thereby preventing the emotional interference that would otherwise disrupt or delay their behavioral responses. ERP results revealed a clear dissociation: higher narcissism significantly predicted reduced amplitudes of the N170, P2, and EPN components in response to violent words. These findings provide neural evidence that narcissistic defense operates during the early stages of information encoding rather than late-stage semantic integration.

### 4.1. Early Neural Defense and Self-Protection

The reduction in N170 and P2 amplitudes suggests a decrease in early sensory evaluation and attentional orienting toward identity-threatening stimuli. Our findings align with Morf and Rhodewalt’s (2001) model, showing that this avoidance is initiated as early as 150–250 ms. Notably, the interaction effect on the P2 in the LDT indicates that this response is specific to violent words. This specificity is further reinforced by our correlation analysis using P2 difference scores, which revealed that higher narcissistic traits are associated with a greater reduction in neural response specifically toward threat relative to the neutral baseline. This effectively rules out the possibility of a general diminished responsiveness to verbal cues, pointing instead to a targeted early filtering of identity-threatening information.

While prior research noted that narcissists exhibit selective attention—such as heightened vigilance toward success-related words (Gu et al., 2013) or the avoidance of first-person pronouns during threat (Marchlewska & Cichocka, 2017)—our data demonstrate that this avoidance manifests as a systematic reduction in neural engagement. By minimizing early threat encoding while preserving stimulus awareness, this mechanism helps protect the self-concept against verbal aggression—aligning with the observed avoidance of self-threatening emotional information (Jauk et al., 2024).

It should be noted that while our tasks utilized isolated violent words rather than real-time interpersonal interactions, these linguistic stimuli were specifically selected to embody the core of verbal violence—attacks on personal dignity and worth (Aytac et al., 2011). In the context of narcissism, the semantic content of such words acts as an acute ego-threat, triggering the same defensive mechanisms used in face-to-face verbal aggression. However, future studies could employ more ecologically valid paradigms, such as simulated social rejection or live verbal feedback, to further validate these findings.

### 4.2. Dimensional Contributions: Vulnerability vs. Grandiosity

In addition to the general effect of total narcissism, a critical finding of this study is the distinct predictive power of the vulnerability dimension within the emotional Stroop task. Regression analyses showed that higher vulnerability specifically predicted reduced EPN and P2 amplitudes during the emotional Stroop task.

Crucially, this neural pattern was mirrored in the behavioral performance: higher scores in narcissistic vulnerability were significantly correlated with a greater decrease in accuracy for violent words relative to neutral ones. This suggests that the observed neural attenuation is not an isolated physiological phenomenon but carries functional consequences. The drop in accuracy indicates that while preemptive cognitive avoidance—manifested as reduced EPN and P2 amplitudes—serves to shield the self from emotional threat, it simultaneously disrupts the allocation of cognitive resources, leading to increased interference during task performance.

According to the Narcissism Spectrum Model, although both dimensions share a core of entitlement, they differ fundamentally in their self-regulatory styles. Vulnerable narcissism is characterized by emotional susceptibility and hypervigilance to criticism (Miller et al., 2017). Our results suggest a paradoxical defense mechanism: to manage high sensitivity, vulnerable individuals may employ preemptive cognitive avoidance or “emotion suppression” (Loeffler et al., 2020), manifesting as smaller EPN amplitudes. This indicates that their characteristic sensitivity leads to a quick reduction in attention to prevent being overwhelmed by emotions. In contrast, individuals high in grandiosity may rely on more active, externalizing defenses that do not require the same early-stage sensory gating.

### 4.3. The “Self-Referential Bias” in Neutral Stimuli

Interestingly, women with narcissistic traits also showed reduced N170 and EPN amplitudes for certain neutral words. This indicates that higher narcissism levels predict attenuated processing of stimuli that lack self-relevance. This may reflect a self-referential bias, wherein narcissistic individuals prioritize self-relevant information over neutral content (Krusemark et al., 2015; Pool et al., 2016). This suggests a calibrated adjustment in processing: the brain focuses on self-protection by reducing early threat encoding while simultaneously devaluing information that lacks emotional or self-referential significance.

### 4.4. Integration of Gender and Socio-Cultural Perspectives

The observed defensive pattern may be linked to gender-socialized coping strategies. Previous research suggests that women often manage to maintain task performance when exposed to emotional stimuli by utilizing efficient neural regulation (J. Yang et al., 2010). In social contexts, women are frequently encouraged to employ emotional restraint and cognitive avoidance to preserve psychological resources (Craske, 2003). In our study, the early-stage neural blunting allowed narcissistic women to maintain task accuracy despite the presence of threatening stimuli. This indicates that “neural avoidance” is a highly efficient way for women with vulnerable narcissistic traits to navigate social threats without disrupting overt behavior.

### 4.5. Limitations and Future Directions

The present study has several limitations that provide directions for future research. First, a primary limitation is the exclusive focus on female university students. While our sample size (N = 70) provided sufficient statistical power to detect the reported neural effects, the use of a non-randomized convenience sample of students limits the generalizability of these findings to a broader population. Furthermore, without a male control group, it remains possible that the observed neural avoidance reflects general cognitive processing styles among university students rather than mechanisms unique to women. Future research should include male cohorts and diverse age groups to clarify the gender-specificity and cross-demographic stability of these early defensive responses.

Second, as a cross-sectional correlation analysis, the results reflect statistical associations between variables rather than definitive causal mechanisms. While we interpret the reduced ERP amplitudes as “defensive strategies,” these pathways require verification through longitudinal designs or experimental manipulations of ego-threat levels.

Third, the generalizability of our findings is constrained by the nature of the experimental tasks. While the lexical decision and emotional Stroop tasks effectively capture early cognitive responses to verbal violence, they represent relatively controlled, laboratory-based conditions. Future studies should incorporate more ecologically valid paradigms, such as real-time social interaction tasks or situational simulations, to better reflect how these defenses operate in daily life.

Fourth, the stimulus materials were restricted to written verbal violence. Since real-world aggression is often multimodal, including auditory clips or visual social scenes would enhance the ecological validity of the research. Additionally, the homogeneous cultural background of our participants precludes cross-cultural comparisons; whether Eastern and Western narcissistic individuals exhibit similar cognitive defenses against verbal violence remains an open question.

Finally, while this study focused on early ERP components (N170, P2, EPN) to identify the “timing” of defense, the role of later components in processing verbal violence warrants further investigation. In particular, further research could explore the neural mediators that link early-stage sensory gating to the subsequent behavioral interference (e.g., accuracy drops) observed in this study. Combining ERPs with other methodologies, such as fMRI for spatial localization or eye-tracking for attentional patterns, would provide a more comprehensive view of these psychological mechanisms.

## 5. Conclusions

This study demonstrates that narcissistic traits in women are associated with defensive cognitive processing of verbal violence. While narcissism showed no significant effect on reaction time, further analysis revealed a task-specific behavioral interference in the emotional Stroop task, where higher vulnerability was associated with a relative drop in accuracy for violent words. Crucially, ERP results revealed a consistent neural dissociation across both tasks, showing that higher PNI total scores predicted smaller amplitudes in early components—specifically the N170 and P2. These results indicate that narcissistic individuals engage in a reduction in attentional and emotional involvement during the early stages of processing. While the vulnerability dimension further modulated neural responses in the emotional Stroop task, the overall findings suggest that for narcissistic women, early-stage cognitive avoidance serves as a primary strategy to protect the self-concept from identity-based aggression, which may occasionally manifest as cognitive interference in demanding behavioral tasks.

## Figures and Tables

**Table 1:** Descriptive Statistics and Normality Tests of PNI (N = 70).

PNI Factors	Mean (n = 70)	SD	Shapiro–Wilk
CSE	39.2	8.51	0.973
EXP	14.5	3.77	0.978
SSSE	21.6	3.68	0.988
HS	25.5	4.93	0.955 *
GF	26.6	6.28	0.977
ER	25.3	5.65	0.966
DEV	23.1	5.86	0.980
NG	60.9	11.4	0.964 *
NV	111.5	20.1	0.976
PNI Total	171.2	29.8	0.986

Note. CSE = Contingent Self-Esteem; EXP = Exploitativeness; SSSE = Self-Sacrificing Self-Enhancement; HS = Hiding the Self; GF = Grandiose Fantasy; DEV = Devaluing; ER = Entitlement Rage; NG = Narcissistic Grandiosity; NV = Narcissistic Vulnerability. * *p* < 0.05.

**Table 2:** Linear Mixed Model Results for ERP Measures in the Lexical Decision Task.

	Predictor	Estimate (β)	Std. Error	t	*p*
N170	(Intercept)	−9.48	1.79	−5.29	<0.001
	PNI	0.03	0.01	2.60	0.011
	Word Type	−0.80	0.07	−1.22	0.228
	PNI × Word Type	0.003	0.004	0.89	0.375
EPN	(Intercept)	−6.50	1.54	−4.21	<0.001
	PNI	0.02	0.01	1.63	0.108
	Word Type	0.18	0.66	0.27	0.785
	PNI × Word Type	−0.004	0.004	−1.03	0.307
P2	(Intercept)	4.31	0.75	5.78	<0.001
	PNI	−0.01	0.004	−2.25	0.028
	Word Type	0.93	0.36	2.61	0.011
	PNI × Word Type	−0.004	0.002	−2.01	0.048
N400	(Intercept)	−0.46	0.92	−0.49	0.623
	PNI	0.004	0.01	0.77	0.445
	Word Type	1.10	0.43	2.57	0.013
	PNI × Word Type	−0.004	0.002	−1.70	0.094

**Table 3:** Linear Mixed Model Results for ERP Measures in the Emotional Stroop Task.

	Predictor	Estimate (β)	Std. Error	t	*p*
N170	(Intercept)	−3.25	3.55	−0.92	0.363
	PNI	0.01	0.02	0.49	0.629
	Word Type	−0.16	0.76	−0.20	0.839
	PNI × Word Type	0.002	0.004	0.34	0.732
EPN	(Intercept)	−6.56	1.28	−5.11	<0.001
	PNI	0.02	0.001	2.97	0.004
	Word Type	−0.47	0.33	−1.46	0.150
	Narcissism × Word Type	0.002	0.002	1.14	0.260
P2	(Intercept)	4.56	0.99	4.62	<0.001
	PNI	−0.01	0.001	2.03	0.046
	Word Type	0.32	0.24	1.38	0.174
	PNI × Word Type	−0.001	0.001	−0.79	0.431
N400	(Intercept)	−3.25	3.55	−0.92	0.363
	PNI	0.01	0.02	0.49	0.629
	Word Type	−0.16	0.76	−0.20	0.839
	PNI × Word Type	0.002	0.004	0.34	0.732

**Table 4:** Pearson correlations between narcissism dimensions (total score, grandiosity, vulnerability) and ERP components (N170, EPN, P2, N400) in the two tasks.

	Grandiosity	Vulnerability	PNI Scores
Lexical Decision Task			
N170-VW	0.31 *	0.29 *	0.31 **
N170-NW	0.21	0.26 *	0.26 *
EPN-VW	0.10	0.13	0.13
EPN-NW	0.17	0.25 *	0.23
P2-VW	−0.24 *	−0.33 **	−0.31 **
P2-NW	−0.03	−0.21	−0.15
N400-VW	−0.01	0.002	−0.002
N400-NW	0.17	0.16	0.17
Emotional Stroop Task			
N170-VW	0.33 **	0.39 ***	0.39 ***
N170-NW	0.29 *	0.35 **	0.35 **
EPN-VW	0.25 *	0.37 **	0.35 **
EPN-NW	0.22	0.34 **	0.31 **
P2-VW	−0.15	−0.29 *	−0.25 *
P2-NW	−0.14	−0.24 *	−0.21
N400-VW	0.11	0.03	0.06
N400-NW	0.07	0.03	0.05

Note. VW = violent words, NW = neutral words; * *p* < 0.05, ** *p* < 0.01, *** *p* < 0.001.

**Table 5:** Hierarchical regression results for three ERP components (N170, EPN, P2) elicited by violent words: Predictions from narcissism total score and dual-dimension models across tasks.

			b (SE)	95%CI	*t*	*p*	β
Lexical Decision Task							
N170	Model1	Grandiosity Vulnerability	0.051 (0.047) 0.020 (0.027)	[−0.043, 0.144] [−0.034, 0.073]	1.09 0.73	0.28 0.47	0.200 0.135
	Model 2	Total scores	0.030 (0.011)	[0.008, 0.058]	2.70	0.009	0.312
EPN	Model 1	Grandiosity Vulnerability	−0.003 (0.042) 0.018 (0.024)	[−0.087, 0.080] [−0.029, 0.066]	0.09 0.76	0.93 0.44	−0.017 0.147
	Model 2	Total scores	0.012 (0.010)	[−0.009, 0.031]	1.06	0.29	0.127
P2	Model 1	Grandiosity Vulnerability	−0.003 (0.021) 0.012 (0.047)	[−0.039, 0.046] [−0.047, 0.001]	0.16 1.29	0.88 0.06	0.029 −0.347
	Model 2	Total scores	−0.014 (0.005)	[−0.024, −0.004]	2.71	0.008	−0.312
Emotional Stroop Task							
N170	Model 1	Grandiosity Vulnerability	0.014 (0.045) 0.050 (0.026)	[−0.077, 0.104] [−0.002, 0.101]	0.31 1.94	0.76 0.06	0.055 0.347
	Model 2	Total scores	0.037 (0.011)	[0.016, 0.059]	3.45	<0.001	0.386
EPN	Model 1	Grandiosity Vulnerability	−0.017 (0.033) 0.046 (0.019)	[−0.082, 0.049] [−0.009, 0.083]	0.51 2.46	0.61 0.02	−0.093 0.444
	Model 2	Total scores	0.024 (0.008)	[0.008, 0.040]	3.05	0.003	0.347
P2	Model 1	Grandiosity Vulnerability	0.024 (0.024) −0.032 (0.014)	[−0.024, 0.073] [−0.060, −0.005]	0.99 2.32	0.32 0.02	0.184 −0.431
	Model 2	Total scores	−0.013 (0.006)	[−0.024, −0.001]	2.14	0.036	−0.251

Neutral words: PNI scores negatively predicted N170 amplitudes in both tasks: Emotional Stroop: b(SE) = 0.033 (0.011), 95% CI [0.011, 0.054], t = 3.06, *p* = 0.003, β = 0.348; Lexical decision: b(SE) = 0.023(0.011), 95% CI [0.002, 0.045], t = 2.17, *p* = 0.03, β = 0.255.

## Data Availability

The data supporting the findings of this study are available from the corresponding author upon reasonable request.

## Supplementary Materials

The following supporting information can be downloaded at: https://www.mdpi.com/article/10.3390/bs16020270/s1, Figure S1: EEG_128_channel_array_map.
