# Supplementary figures and images for: Vulnerable Narcissism Modulates Early Neural Processing of Verbal Violence in Women: An ERP Study
Source: Behav Sci (Basel). 2026 Feb 12;16(2):270. doi: 10.3390/bs16020270 (PMC12938278; doi:10.3390/bs16020270)

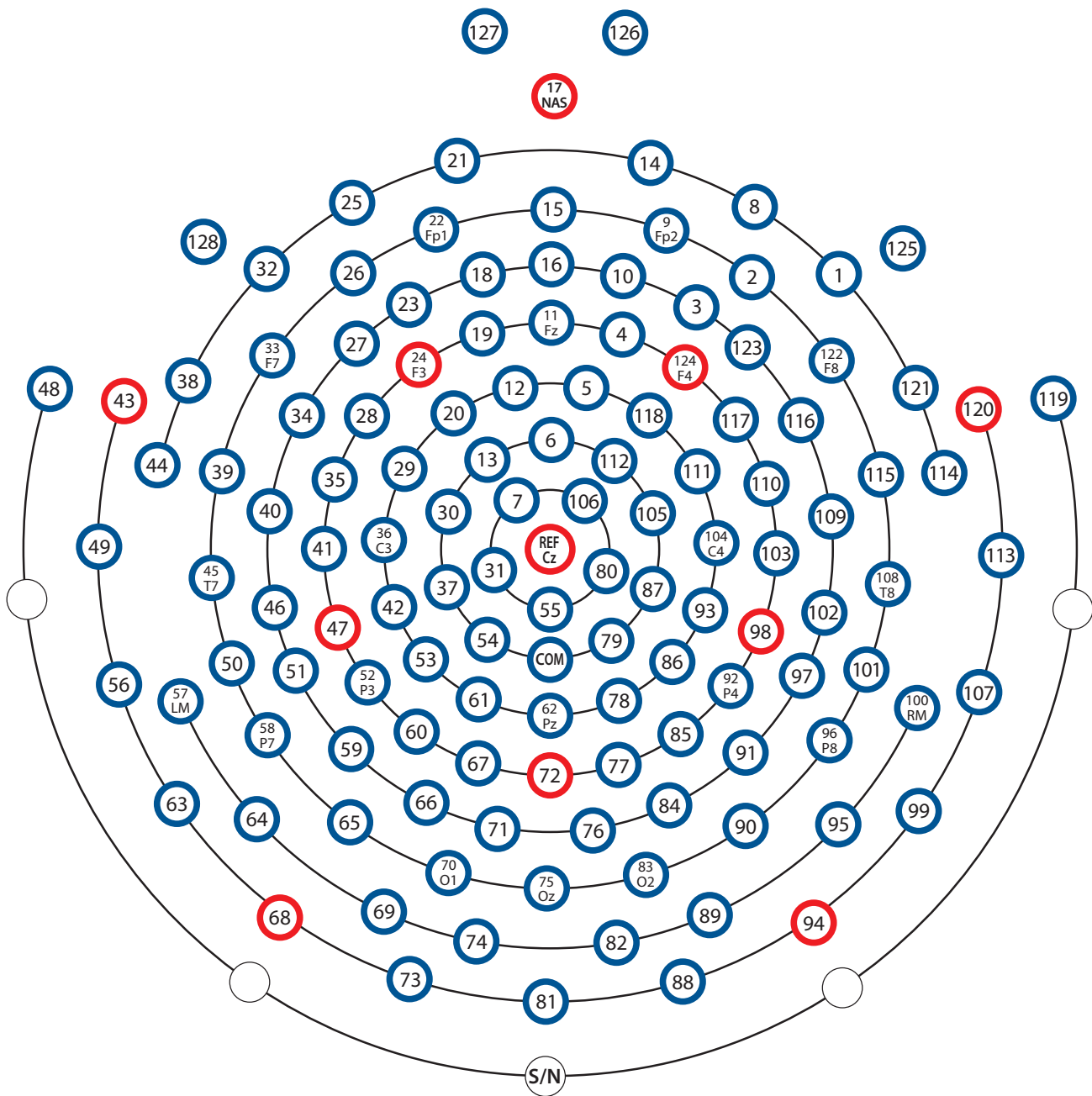

Supplement: Supplementary file 1 [file behavsci-16-00270-s001.zip › behavsci-4106869-supplementary.pdf]
